# Proenkephalin A 119-159 in Kidney Transplantation: A Novel Biomarker for Superior Tracking of Graft Function Trajectories

**DOI:** 10.3389/ti.2025.14366

**Published:** 2025-05-22

**Authors:** Louise Benning, Marvin Reineke, Camila Eleuterio Rodrigues, Florian Kälble, Claudius Speer, Claudia Sommerer, Christoph F. Mahler, Felix C. F. Schmitt, Markus Mieth, Martin Zeier, Christoph Michalski, Arianeb Mehrabi, Oliver Hartmann, Markus Zorn, Sophie C. Anker, David Czock, Markus A. Weigand, Zoltan Endre, Christian Morath, Christian Nusshag

**Affiliations:** ^1^ Department of Nephrology, Heidelberg University Hospital, Medical Faculty, Heidelberg University, Heidelberg, Germany; ^2^ Department of Nephrology, Prince of Wales Hospital, Sydney, NSW, Australia; ^3^ Department of Nephrology, Hospital das Clínicas - University of São Paulo School of Medicine, São Paulo, Brazil; ^4^ Department of Anaesthesiology, Heidelberg University Hospital, Medical Faculty, Heidelberg University, Heidelberg, Germany; ^5^ Department of General, Visceral, and Transplantation Surgery, Heidelberg University Hospital, Medical Faculty, Heidelberg University, Heidelberg, Germany; ^6^ SphingoTec GmbH, Berlin, Germany; ^7^ Central Laboratory of University Hospital Heidelberg, Department of Endocrinology and Metabolism, Heidelberg University Hospital, Medical Faculty, Heidelberg University, Heidelberg, Germany; ^8^ Department of Clinical Pharmacology and Pharmacoepidemiology, Heidelberg University Hospital, Medical Faculty, Heidelberg University, Heidelberg, Germany

**Keywords:** delayed graft function, proenkephalin A, risk stratification, graft function trajectory, study enrichment, slow graft function, kidney graft recovery

## Abstract

Accurate assessment of graft function trajectories after kidney transplantation is essential for optimizing patient management. Slow graft function (SGF) and delayed graft function (DGF) are associated with impaired recovery, yet current diagnostic tools lack granularity for timely risk stratification. Proenkephalin A 119-159 (penKid) may improve graft function assessment, enhancing risk stratification for SGF, DGF, and associated outcomes. This prospective study evaluated 159 kidney transplant recipients at Heidelberg University Hospital to compare plasma penKid levels with current risk-indicators for poor (functional) graft trajectories. Validation was conducted using an independent transplant cohort from Sydney. Clinical relevance of biomarker-indicated changes in graft function was assessed using multivariable regression models and AUROC analyses. From day one post-transplant, penKid outperformed serum creatinine (SCr) in identifying functional trajectories associated with DGF (AUROC penKid: 0.87 vs. SCr: 0.56) and differentiated SGF from DGF (AUROC penKid: 0.79 vs. SCr: 0.33) up to eight days earlier. PenKid further demonstrated superior granularity in assessing DGF severity and 30-day outcomes. After adjustment for common risk factors, penKid remained the strongest risk stratifier for all tested outcomes. PenKid is a superior biomarker for earlier assessment of graft function trajectories, offering potential to enhance personalized care and clinical trial designs in kidney transplantation.

## Graphical Abstract

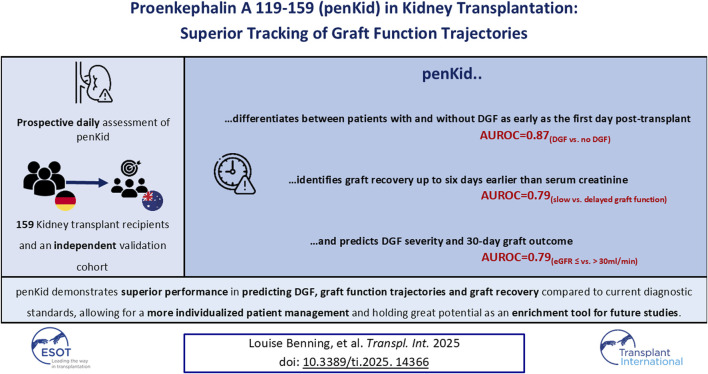

## Introduction

Early and accurate discrimination between diverse graft function trajectories following kidney transplantation is essential for individualized patient management. Utilizing appropriate diagnostic tools can enable timely risk assessment for adverse outcomes, such as delayed graft function (DGF) and its severity, thereby supporting informed clinical decision-making. DGF is a common complication after kidney transplantation, with reported incidences ranging from 5% to 50% [1–4]. Early identification of functional trajectories at risk for DGF is therefore of critical importance. Especially, prolonged DGF has been shown to negatively impact one-year graft function and long-term graft survival [5–14].

DGF is typically defined as the requirement for kidney replacement therapy (KRT) within the first week post-transplantation [5, 9]. However, this definition is inherently limited due to its dependence on subjective clinical judgment and variability in institutional protocols regarding the initiation of KRT apart from emergency criteria. Moreover, it lacks granularity, as it encompasses a wide range of indications for KRT, from transient issues such as hyperkalemia to more severe conditions like critical hypervolemia, vascular complications, metabolic disturbances, and rejection episodes [5, 9, 15]. As of today, the severity of DGF and its complications can only be retrospectively defined.

The optimal clinical management, such as the start of KRT (in the absence of emergency criteria) or the timing of kidney biopsies is particularly hindered by the absence of timely and accurate tools for assessing critical graft function trajectories at risk using current diagnostic standards. Especially in patients without immediate graft function (IGF), evaluating graft function trajectories remains largely speculative and is typically based on clinical experience, incorporating donor criteria and postoperative trends in serum creatinine (SCr) or urine output. However, the slow and insensitive kinetics of SCr, the weak correlation between urine output and kidney function, and the influence of non-renal factors on SCr levels - such as KRT, muscle mass, and medication - further complicate the assessment [16–18].

These limitations likewise impede the development of new therapeutic strategies and the establishment of appropriate inclusion criteria for interventional trials. Consequently, there is a pressing need for more reliable biomarkers to enable early and accurate assessment of graft function trajectories, thereby optimizing patient care and therapeutic approaches in high-risk populations.

Recently, proenkephalin A 119-159 (penKid) has emerged as a novel biomarker that may more adequately reflect kidney function, particularly in critically ill patients with acute kidney injury (AKI) and under non-steady state conditions [19, 20]. penKid is a byproduct derived from the breakdown of the same precursor molecule as endogenous opioids, called enkephalins [21]. With its small molecular mass (4.5 kDa), penKid appears to be freely filtered through the glomerulus with no evidence of protein binding [19], rendering it a biomarker for assessing kidney functional integrity.

Given the pathophysiological similarities between cold ischemia (CIT)-induced injury in transplanted kidneys and ischemia-reperfusion injury (IRI) in native kidneys [3, 22], we hypothesize that penKid could enable earlier and more robust differentiation of individual graft function trajectories and their associated outcomes. Such capabilities could significantly enhance risk stratification and clinical decision-making in the post-transplant setting, paving the way for improved patient outcomes and interventional trials aimed at mitigating DGF in the future.

## Materials and Methods

### Study Design

Between November 2021 and July 2023, this prospective, single-center, real-world study at Heidelberg University Hospital quantified daily plasma penKid levels on weekdays in 159 consecutive kidney transplant recipients, from admission to discharge (Heidelberg study). The study was part of the PARTICIPATE study, evaluating the diagnostic utility of penKid in routine clinical practice across various settings. It was approved by the University of Heidelberg ethics committee and registered in the German Clinical Trials Register (DRKS00026776). Patient consent was waived as penKid assessment was integrated into routine diagnostics, imposing no additional burden. The reliability of penKid kinetics and diagnostic performance was validated in an independent Sydney cohort, with pre- and first post-transplant day data analyzed. This study was approved by the South Eastern Sydney Local Health District Human Research Ethics Committee (2021/ETH11450) and registered in the Australian New Zealand Clinical Trials Registry. Both studies adhered to the Principles of the Declaration of Istanbul on Organ Trafficking and Transplant Tourism and the Declaration of Helsinki.

### Quantification of Proenkephalin A 119-159

PenKid was quantified in EDTA plasma using the sphingotest^®^ penKid^®^ immunoassay from SphingoTec GmbH (Hennigsdorf, Berlin), as described previously [23].

### Definition of Transplant-Related Outcomes

In alignment with previous DGF biomarker studies [10, 24], recovery of graft function in patients without DGF was additionally classified in slow graft function (SGF) and immediate graft function (IGF). SGF and IGF were distinguished using a SCr reduction ratio (difference between the initial SCr collected within an hour of transplantation and the SCr on day 7 divided by the initial SCr) of <0.7 and ≥0.7, respectively [10]. DGF was primarily defined as the necessity for KRT within the initial 7 days post-transplant, aligning with the widely adopted definition for DGF. The KRT indication was made by the respective treating physician. Considering the significant duration-dependent negative impact of prolonged DGF [8, 9, 11–13], we categorized the severity of DGF for further analysis as follows: (1) No DGF (primary graft function); (2) KRT only within the first 24 h (mild DGF); (3) KRT up to Day 7 post-transplant (moderate DGF); and (4) KRT required beyond Day 7 post-transplant (severe DGF). Poor 30-day graft outcome was defined as eGFR ≤30 mL/min/1.73 m^2^ using the CKD-EPI equation.

### Statistics

Quantitative data are reported as median with interquartile range (IQR). Group comparisons for continuous variables used the Kruskal-Wallis test, while categorical data were analyzed with Pearson’s Chi-squared Test. Biomarker data were log-transformed. Receiver-operating-characteristic (ROC) curves assessed sensitivity and specificity, with the area under the ROC (AUROC) used to compare predictive accuracy. To assess penKid’s independence from other variables (e.g., cold ischemia time, recipient KRT vintage, transplant modality, donor age, and donor SCr), likelihood ratio chi-square tests were applied to nested multivariable logistic regression models for DGF, comparison of SGF versus DGF and 30-day graft outcomes. To determine which factors influence absolute penKid concentrations in patients prior to transplantation (pre-Tx) or changes in penKid levels after transplantation (d0/d1), two linear regression models were performed for pre-transplant penKid levels (including the variables KRT duration pre-transplant, age, diabetes, body mass index, congestive heart failure, sex, adipositas, hypertension and peripheral artery disease) and penKid changes from pre-transplant to first post-transplant day (including the variables donor modality, donor age, donor SCr, CIT, and KRT duration pre transplant). For continuous variables, odds ratios (OR) were standardized to describe the OR for a change of one IQR. Cases missing penKid or SCr data were excluded. All statistical tests were two-tailed, with significance set at P < 0.05. Analyses were conducted using R version 4.2.2 (libraries: rms, Hmisc, ROCR) and SPSS version 22.0 (SPSS Inc., Chicago, IL, USA).

## Results

### Study Cohort

Between November 2021 and July 2023, a total of 159 kidney transplant recipients were consecutively enrolled in the Heidelberg study. Baseline characteristics and outcomes for patients with and without DGF are summarized in Table 1. Recipients with DGF were generally older, male, had higher body mass index (BMI), and a longer KRT vintage. They were also more likely to have received organs from male donors or donors with a history of arterial hypertension. In addition, recipients with DGF more frequently received transplants from deceased donors and experienced longer CIT compared to those without DGF. Length of hospital stay post-transplantation was longer for DGF patients. At discharge, both SCr and penKid levels were significantly higher in patients with DGF compared to patients without DGF. No significant differences were observed between groups regarding donor age, donor SCr, history of diabetes mellitus, number of previous transplants, type of induction therapy, or complement-dependent cytotoxicity analysis.

**TABLE 1:** Baseline characteristics.

Variable	All N = 159	No DGF N = 106	DGF N = 53	*P*-value
Recipient				
Age (years), median (IQR)	49 (39–60)	47 (36–58)	53 (46–61)	0.02
Sex (female), N (%)	72 (45.3)	55 (51.9)	17 (32.1)	0.03
BMI (kg/m^2^), median (IQR)	25.0 (22.5–29.0)	24.0 (21.7–26.8)	26.9 (24.4–30.9)	<0.001
Dialysis Vintage (years), median (IQR)	6.5 (2.2–9.0)	4.8 (1.1–8.3)	8.0 (5.0–9.5)	<0.001
Donor				
Age (years), median [IQR]	55 (46–62)	55 (45–62)	56 (48–61)	0.90
Sex (female), N (%)	74 (46.5)	58 (54.7)	18 (34.0)	0.01
Hypertension, N (%)	44 (28.8)	22 (21.6)	22 (43.1)	0.01
Diabetes, N (%)	6 (3.9)	2 (2.0)	4 (7.8)	0.19
S-Creatinine (mg/dL), median (IQR)	0.8 (0.7–1.0)	0.8 (0.7–0.9)	0.9 (0.7–1.3)	0.09
Transplant-Related				
Transplant Modality Living, N (%) Deceased, N (%)	50 (31.4) 109 (68.6)	48 (45.3) 58 (54.7)	2 (3.8) 51 (96.2)	<0.001
Number of Transplants First, N (%) Retransplants, N (%)	140 (88) 19 (12)	97 (92) 9 (8)	43 (95) 10 (19)	0.07
Cold Ischemia Time (hours), median (IQR)	10.0 (2.5–14.1)	7.7 (2.0–13.3)	12.7 (9.9–16.2)	<0.001
Median HLA (A, B, DR) Mismatches (IQR)	3 (2–4)	3 (2–4)	3 (2–4)	0.49
Complement-dependent cytotoxicity (panel reactivity of >30%), N (%)	28 (17.6)	16 (15.1)	12 (22.6)	0.34
Induction Therapy Rituximab, N (%) Anti-thymocyte globulin, N (%) Interleukin-2 receptor antagonist, N (%) Other, N (%)	8 (5.0) 34 (21.4) 116 (73.0) 8 (5.0)	6 (5.7) 18 (17.0) 80 (75.5) 8 (7.5)	2 (3.8) 16 (30.2) 36 (67.9) 0 (0)	0.90 0.09 0.41 0.10
Short-Term Outcomes				
Length of Stay (days), median (IQR)	16.0 (12.0–21.5)	13.5 (12.0–17.0)	23.0 (18.0–31.0)	<0.001
S-Creatinine at Discharge (mg/dL), median (IQR)	1.7 (1.3–2.4)	1.4 (1.2–1.8)	2.5 (1.8–3.7)	<0.001
penKid at Discharge (pmol/L), median (IQR)	117.1 (87.2–149.5)	109.1 (80.9–133.8)	146.7 (115.0–242.1)	<0.001

BMI, body mass index; DGF, delayed graft function; HLA, human leucocyte antigen; IQR, interquartile range; N, number; penKid, Proenkephalin A 119-159.

### Assessment of Graft Function Trajectory

**FIGURE 1:**
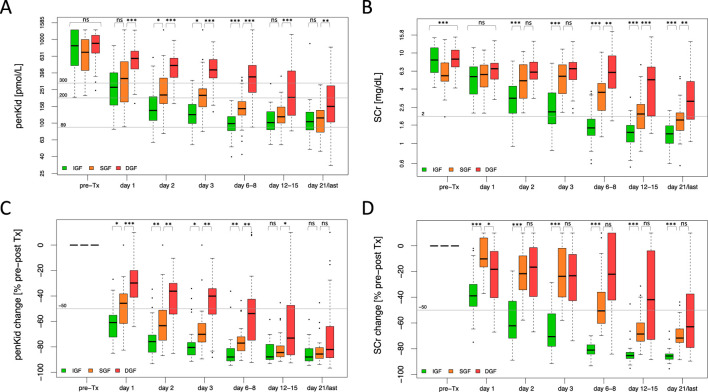
Biomarker trajectory of proenkephalin A 119-159 and serum creatinine to discriminate critical scenarios of graft function recovery. **(A, B)** Absolute biomarker trajectories until patient discharge stratified by recovery of graft function. **(C, D)** Relative biomarker changes until patient discharge comparing pre-transplant biomarker levels to the respective post-transplant days stratified by recovery of graft function. IGF (green): N = 61, SGF (orange): N = 45, DGF (red): N = 53. Data are reported as box-and-whisker plots (interquartile range, minimum to maximum). The grey lines indicate penKid cut-offs at 300 pmol/L, 200 pmol/L and 89 pmol/L (the last being the upper reference limit for healthy individuals) **(A, B)**, or a 50% decrease cut-off compared to pre-transplant biomarker levels **(C, D)**. For SCr, the grey line signifies an SCr of 2 mg/dL for orientation. Both y-axes are log-transformed. d, days; DGF, delayed graft function; IGF, immediate graft function; penKid, Proenkephalin A 119-159; SCr, serum creatinine; SGF, slow graft function; Tx, transplant. *P ≤ 0.05, **P ≤ 0.01, ***P ≤ 0.001. NS, *P* > 0.05.

Considering the variation in the scenarios of graft function trajectory beyond DGF, a SCr reduction ratio, calculated between the SCr pre-transplant and the SCr on day 7 post-transplant, was additionally employed to differentiate between slow (SGF), immediate (IGF) graft function and DGF. As shown in Figure 1, pre-transplant penKid levels did not differ in relation to the graft function trajectory, whereas SCr showed significant differences; but this rather determined by the timing of last KRT rather than true differences in kidney function pre-transplant. Absolute penKid levels and particularly changes from baseline provided robust diagnostic performance from day 1 post-transplant, distinguishing IGF, SGF, and DGF. In contrast, SCr only began to differentiate between SGF and DGF on days 6–8 (Figures 1A–D).

**FIGURE 2:**
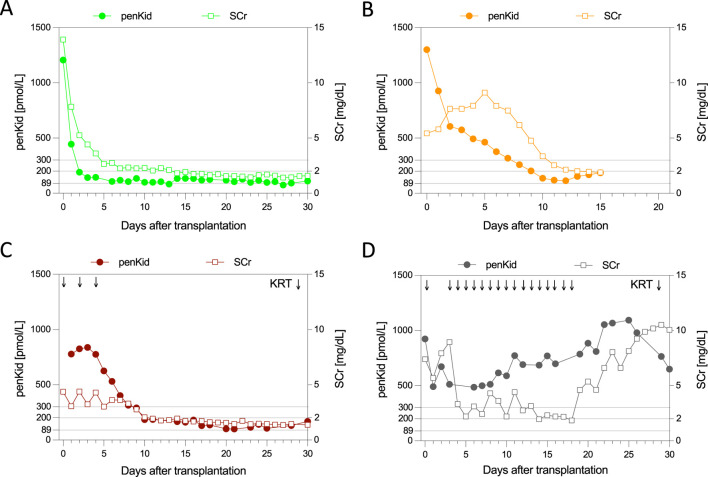
Individual biomarker trajectories of proenkephalin A 119-159 and serum creatinine identify four outcome scenarios. Patient with primary/immediate graft function **(A)**, patient with slow graft function **(B)**, patient with DGF and favorable 30d-graft outcome **(C)**, and a patient with DGF and poor 30-d graft outcome **(D)**. The grey lines indicate penKid cut-offs at 300 pmol/L, 200 pmol/L, and 89 pmol/L (the last being the upper reference limit for healthy individuals). For SCr, the grey line signifies an SCr of 2 mg/dL for orientation. Both y-axes are log-transformed. KRT, kidney replacement therapy; penKid, Proenkephalin A 119-159; SCr, serum creatinine.

Individual patient trajectories (Figures 2A–D) further highlighted penKid’s superiority and time advantage over SCr. Based on longitudinal data, four outcome scenarios were identified: primary graft function (immediate decline in both biomarkers, Figure 2A), SGF (no KRT, immediate decline in penKid but not SCr, Figure 2B), moderate DGF severity with favorable outcomes (KRT, elevated SCr, earlier penKid decline, Figure 2C), and severe DGF severity with poor outcomes (KRT, persistent elevation of both markers, Figure 2D). Notably, unlike SCr, penKid levels were unaffected by KRT, as shown in Figures 2C,D.

### Assessing the Severity of Delayed Graft Function

**FIGURE 3:**
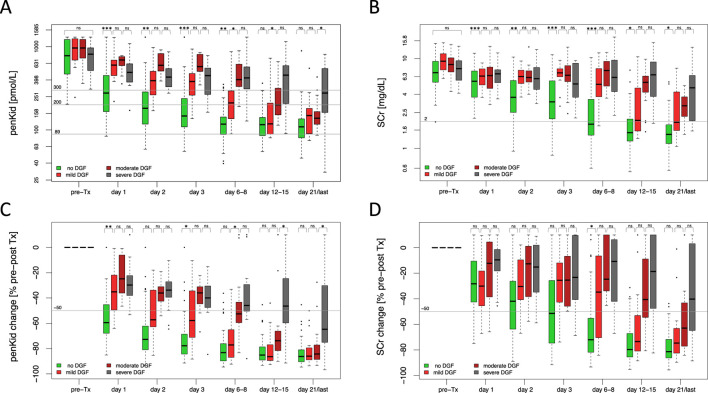
Overall biomarker trajectories of proenkephalin A 119-159 and serum creatinine in relation to severity of delayed graft function. **(A, B)** Absolute biomarker trajectories until discharge stratified by DGF severity. **(C, D)** Relative biomarker changes until patient discharge comparing pre-transplant biomarker levels to the respective post-transplant days stratified by DGF severity. No DGF (green): N = 106, mild DGF (red): N = 19, moderate DGF (dark red): N = 17, severe DGF (grey): N = 17. Data are reported as box-and-whisker plots (interquartile range, minimum to maximum). The grey lines indicate penKid cut-offs at 300 pmol/L, 200 pmol/L, and 89 pmol/L (the last being the upper reference limit for healthy individuals) **(A, B)**, or a 50% decrease cut-off compared to pre-transplant biomarker levels **(C, D)**. For SCr, the grey line signifies an SCr of 2 mg/dL for orientation. Both y-axes are log-transformed. d, days; DGF, delayed graft function; KRT, kidney replacement therapy; penKid, Proenkephalin A 119-159; SCr, serum creatinine; Tx, transplant. *P ≤ 0.05, **P ≤ 0.01, ***P ≤ 0.001. NS, *P* > 0.05.

As the conventional definition of DGF does not allow to differentiate early from late recovery of graft function after the first KRT was initiated and thus does not reflect the different severity levels of DGF, penKid and SCr levels were also assessed in relation to varying degrees of DGF severity, namely mild DGF, moderate DGF, and severe DGF (Figure 3). In mild DGF, functional improvement was evident by days 6–8 post-transplant, with lower absolute penKid levels and more pronounced changes from baseline compared to moderate DGF (Figures 3A,C). Absolute SCr levels and changes, however, failed to differentiate severity during this timeframe but reflected improvement later, with declines apparent at days 12–15 for mild DGF (Figures 3A–D). Similarly, penKid levels decreased in moderate DGF starting on days 12–15, while SCr showed comparable trends only by day 21.

### Graft Function Trajectory and Its Association to Critical Outcomes

To demonstrate the clinical relevance of early identification of distinct graft function trajectories for critical outcomes, we performed AUROC analyses and multivariate logistic regression models, incorporating established risk factors for poor graft outcomes across various outcome scenarios.

**FIGURE 4:**
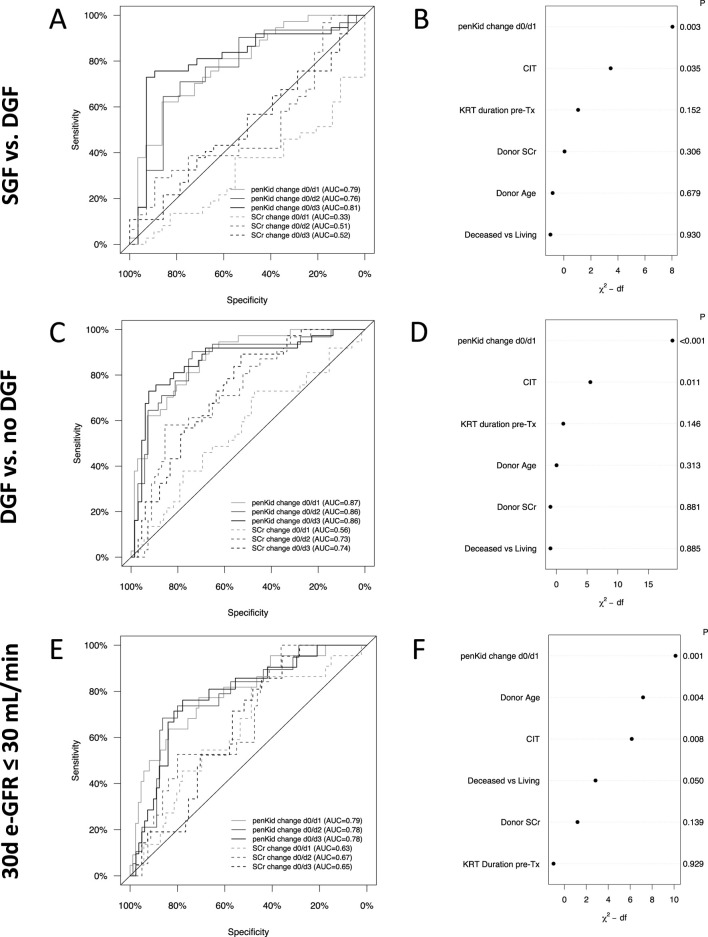
Longitudinal changes of proenkephalin A 119-159 or serum creatinine and their association with critical graft outcomes. Receiver-operating characteristics analysis for relative biomarker change from pre-transplant to first three post-transplant days to distinguish between SGF and DGF **(A)**, DGF and no DGF **(C)** and 30-day graft outcome **(E)**. Multivariate logistic regression model to analyze the value of penKid changes to distinguish SGF from DGF **(B)**, DGF from no DGF **(D)** and 30-day graft outcome **(F)**. IGF: N = 61, SGF: N = 45, DGF: N = 53, no DGF = 106, 30d-eGFR≤30 mL/min: N = 35, 30d-eGFR>30 mL/min: N = 124. AUC, area under the curve; CIT, cold ischemia time; DGF, delayed graft function; eGFR, estimated glomerular filtration rate; IGF, immediate graft function; P, p-value; penKid, Proenkephalin A 119-159; SCr, serum creatinine; SGF, slow graft function; Tx, transplant.

For discriminating SGF from DGF, the AUROC for penKid on day 1 was 0.79 (95% CI 0.68–0.90, *P* < 0.001), while SCr changes never provided significant discrimination ability at that time (change was higher in the SGF group compared to the DGF group) (Figure 4A). PenKid changes continued to outperform SCr changes through days 2 and 3 with an AUROC of 0.76 (95% CI 0.64–0.89, *P* < 0.001) and 0.81 (95% CI 0.70–0.92, *P* < 0.001), respectively. Corresponding AUROCs for SCr were 0.51 (95% CI 0.36–0.66, *P* = 0.539) and 0.52 (95% CI 0.38–0.66, *P* = 0.644) on days 2 and 3, respectively.

Similar patterns were observed for identifying patients with DGF or poor 30-day graft outcome (Figures 4C–F). As early as the first post-transplant day, penKid changes distinguished between patients with and without DGF with an AUROC of 0.87 (95% CI 0.81–0.94, *P* < 0.001), outperforming SCr (AUROC 0.56, 95% CI 0.45–0.68, *P* = 0.332). Comparable performance for penKid was observed on days 2 and 3, with AUROCs of 0.86 (95% CI 0.78–0.94, *P* < 0.001), compared to SCr’s lower AUROCs of 0.73 (95% CI 0.63–0.83, *P* < 0.001) and 0.74 (95% CI 0.64–0.83, *P* < 0.001) (Figure 4C). Even in predictive performance analysis across subpopulations (deceased vs. living, male vs. female, etc.), penKid was a consistent risk stratifier for predicting DGF (Supplementary Figure S1).

Stratifying 30-day graft outcomes by eGFR ≤30 mL/min/1.73 m^2^, penKid changes from pre-transplant to day 1 yielded an AUROC of 0.79 (95% CI 0.69–0.90, *P* < 0.001) for predicting 30-day eGFR ≤30 mL/min/1.73 m^2^, compared to SCr changes with an AUROC of 0.63 (95% CI 0.51–0.76, *P* = 0.080) (Figure 4E).

Multivariate logistic regression analysis revealed that graft function changes indicated by penKid changes were the strongest discriminator for SGF versus DGF (Figure 4F) and strongest predictor for the tested outcomes DGF and eGFR ≤30 mL/min/1.73 m^2^ (Figures 4D,F). Likewise, after adjustment, changes in penKid effectively identified patients with higher risk profiles across different outcome scenarios. Specifically, the OR (per IQR of penKid) for SGF versus DGF was 5.2 (95% CI: 1.8–15.2), for DGF versus no DGF it was 17.3 (95% CI: 5.0–60.6), and for eGFR ≤30 mL/min/1.73 m^2^ it was 4.6 (95% CI: 1.8–11.8). In contrast, when penKid was replaced by SCr in the multivariate model, the ability to stratify risk via a functional biomarker was significantly diminished. The OR (per IQR SCr) for SGF versus DGF, DGF versus no DGF and for eGFR ≤30 mL/min/1.73 m^2^, dropped to 0.3 (95% CI: 0.1–0.8), 1.0 (95% CI: 0.5–2.1) and 1.6 (95% CI: 0.7–3.7), respectively.

Further, linear regression analysis was used to assess the association between penKid levels and recipient- and donor-related factors relevant to transplant outcomes (Supplementary Figure S2). Absolute penKid levels pre-transplant were mainly related to the duration of KRT prior transplantation, but also to recipient age, whereas changes in penKid levels were exclusively associated with donor modality (living vs. deceased donation).

### Cut-Offs to Identify Graft Function Trajectories at Risk for Delayed Graft Function or Poor 30-Day Graft Outcome

To develop a “rule out” test for DGF with >95% sensitivity, a penKid cut-off of >300 pmol/L on day 1 post-transplant achieved 95.1% sensitivity (95% CI 83.9–98.7) and 56.5% specificity (95% CI 46.3–66.2), with an OR of 25.4 (95% CI 5.8–111.3), a PPV of 49.4% and an NPV of 96.3%. For SCr, a cut off of 3.5 mg/dL selected to achieve a comparable sensitivity of 95%, achieved a sensitivity of 95.1% (95% CI 83.9–98.7) and 20.7% specificity (95% CI 13.6–30.0), with an OR of 5.1 (95% CI 1.1–22.9), a PPV of 35.0% and an NPV of 90.5%.

A ≤50% reduction in penKid from pre-transplant to day 1 yielded 89.2% sensitivity (95% CI 75.3–95.7) and 66.7% specificity (95% CI 55.2–76.5) with an OR of 16.5 (95% CI 5.2–52.0). For a ≤50% reduction in SCr, the OR was 1.6 (95% CI 0.4–6.4, p = 0.711).

For predicting 30-day eGFR ≤30 mL/min/1.73 m^2^, a penKid cut-off of >300 pmol/L on day 1 showed 84% sensitivity (95% CI 65.4–93.6) and 45.8% specificity (95% CI 36.7–55.2), with an OR of 4.4 (95% CI 1.4–13.8), a PPV of 26.5% and an NPV of 92.5%. For SCr, a cut off of 3.5 mg/dL achieved 96.0% sensitivity (95% CI 80.5–99.3) and 18.7% specificity (95% CI 12.4–27.1), with an OR of 5.5 (95% CI 0.7–43.2), a PPV of 21.6% and an NPV of 95.2%. A ≤50% reduction in penKid yielded 81.8% sensitivity (95% CI 61.5–92.7) and 55.8% specificity (95% CI 45.3–65.8), with an OR of 5.7 (95% CI 1.8–18.2), a PPV of 32.1% and an NPV of 92.3%. For a ≤50% reduction in SCr, the OR is 3.1 (95% CI 0.4–25.2, p = 0.473).

### Validation in an Independent Transplant Cohort

In the Sydney study, 60 patients were recruited from September 2022 to June 2024. Patient characteristics and biomarker trajectories for penKid and SCr closely resembled those of the Heidelberg study (Supplementary Table S1; Supplementary Figures S3–S5). Extent of penKid changes (d0 vs. d1) correlated with DGF development and 30-day eGFR ≤30 mL/min/1.73 m^2^, achieving an AUROC of 0.88 (95% CI 0.75–1.0, P < 0.001) and 0.82 (95% CI 0.64–0.99, P = 0.007), respectively (Supplementary Figures S3E, S5E). Similar trends in biomarker differentiation for IGF, SGF, and DGF, and comparable diagnostic performance using a penKid cut-off of 300 pmol/L or a 50% reduction rate, were confirmed (Supplementary Figures S3–S5; Supplementary Table S2).

## Discussion

The increasing use of marginal kidneys and implementation of DCD programs to address organ shortages has elevated the incidence of organs without IGF and/or DGF, presenting a significant clinical challenge [1, 3, 25]. Although no effective treatment strategies currently exist, early diagnosis and risk stratification of graft function trajectory are essential for improving individualized care and developing future therapeutic approaches.

This monocentric, prospective study is the first to evaluate the diagnostic value of penKid for assessing and risk stratifying graft function trajectories and outcomes in the immediate postoperative phase following kidney transplantation. penKid demonstrated the ability to provide significantly earlier insights in patients without immediate graft function by differentiating SGF from DGF, up to 6 days prior to detectable improvements in SCr. This early discrimination may facilitate more informed clinical decision-making through a personalized, penKid-guided approach. Specifically, early identification of SGF could allow for the avoidance of unnecessary interventions such as KRT or kidney biopsy, whereas in cases of DGF, earlier initiation of these measures may be justified. The same applies to distinguishing DGF from no DGF, where penKid outperformed SCr and donor criteria as early as the first post-transplant day in total cohort as well as in subgroup analyses, offering a diagnostic time advantage of several days for individual patients.

On the other hand, penKid’s superior granularity in identifying graft function recovery in patients with DGF allows for nuanced sub-classification of DGF severity and associated outcomes, overcoming the limitations of the previously binary DGF definition (DGF versus no DGF) and acknowledging the severity-dependent impact of DGF on long-term outcomes [11–13]. Further, multivariate models incorporating established poor graft outcome risk factors confirmed penKid as the strongest independent risk discriminator of SGF versus DGF, DGF versus no DGF and poor 30-day outcomes, and underlined the independent role of penKid as a marker of kidney integrity by showing a strong association of baseline penKid levels and penKid changes with KRT duration prior transplantation and donor modality, respectively.

Interestingly, our data indicated that, unlike SCr, penKid levels remained remarkably unaffected by KRT, suggesting that penKid may be a more reliable marker of kidney function integrity than SCr during KRT. While we have validated these very unique characteristics in other AKI contexts among critically ill patients [26], further *in-vivo* studies utilizing various KRT techniques are necessary to better understand and confirm these findings. A possible explanation could be a high turnover rate of penKid, characterized by rapid production and metabolism, as it is a small protein with no evidence of protein binding.

Recently, studies have highlighted the considerable potential of penKid in predicting AKI and related outcomes, particularly in critically ill, non-transplanted patients. PenKid has been identified as an early predictor of AKI, an indicator of subclinical AKI [20, 27], and a correlate of GFR and AKI severity. It has also shown promise as a potential risk stratifier for death or the requirement of KRT in clinical contexts such as sepsis and cardiac surgery [27–30]. Consistent with these observations, Beunders et al. demonstrated in a cohort of patients with septic shock that penKid concentrations more accurately reflected measured GFR than traditional estimates of kidney function, such as from endogenous creatinine clearance [31]. The authors further validated a novel penKid-SCr-based GFR equation, showing that this outperformed most creatinine-based equations [32]. Beyond the critical care setting, Schulz et al. found that higher penKid levels were associated with faster kidney function decline and an increased risk of new-onset chronic kidney disease (CKD) over a 16.6-year follow-up in a cohort of 2,568 participants without baseline CKD [33]. This again suggests that penKid may be a more sensitive diagnostic marker for changes in kidney function than SCr.

In the single published study of penKid in kidney transplant recipients, Kieneker et al. found that higher penKid levels were significantly associated with poor long-term outcomes and graft failure in a cohort of 664 recipients, measured, however, at least 1 year post-transplant [34].

In contrast, our present study is the first to investigate penKid as a longitudinal biomarker in the immediate post-transplant period, aiming to predict and stratify different graft function trajectories, and their outcomes. This is particularly important given that despite advances in identifying potential biomarkers for DGF, such as Neutrophil Gelatinase-Associated Lipocalin (NGAL), Kidney Injury Molecule-1 (KIM-1), and plasma cell-free DNA, none have yet been incorporated into clinical practice [21, 35–39]. Further, a key limitation of such damage-based biomarkers is their limited specificity for functional changes or DGF in general, as IRI during kidney retrieval inevitably releases damage-associated molecules [35]. In addition, unlike functional biomarkers such as penKid, damage biomarkers do neither correlate linearly with kidney function impairment nor with recovery or residual kidney function capacity after reperfusion.

Despite these encouraging findings, several limitations need to be addressed. First, although validation was performed using an independent transplant cohort, larger multicenter studies across diverse healthcare systems and organ donation settings are necessary to confirm these results and ensure broader generalizability. Second, as the study design was observational, it remains speculative whether real-time clinical decision-making based on penKid levels could directly improve patient management and outcomes. Nevertheless, penKid-guided risk stratification and diagnostic enrichment could play a pivotal role in optimizing future clinical trial designs, individualized patient management and therapeutic interventions. Lastly, this study did not include a direct comparison of penKid with other established or emerging kidney biomarkers, such as Cystatin C, NGAL, KIM-1 and others. The real-world clinical setting of our study limited the feasibility of incorporating these additional biomarkers. Future research should prioritize head-to-head comparisons between penKid and other kidney biomarkers to clarify their relative accuracy and clinical utility in assessing graft function trajectories and transplant outcomes.

In conclusion, our findings contribute to the growing body of evidence supporting penKid as a superior biomarker reflecting kidney function and integrity, extending clinical utility beyond an established role in predicting (subclinical) AKI and outcomes in critically ill patients. In this real-world post-transplantation setting, penKid demonstrated for the first time robust reliability as a biomarker for distinguishing IGF, SGF, and DGF, assessing DGF severity, and predicting associated 30-day graft outcomes earlier than current clinical standards across two independent transplant cohorts.

Given its high discriminatory power in detecting and sub-characterizing changes in graft function, penKid holds great potential for use in future studies investigating DGF incidence in transplant programs utilizing DCD, in evaluating machine perfusion techniques, or as an enrichment tool for studies evaluating potential therapeutic interventions to mitigate SGF or DGF in the future.

## Data Availability

The data underlying this article will be shared on reasonable request to the corresponding author. Requests to access the datasets should be directed to christian.nusshag@med.uni-heidelberg.de.

## Abbreviations

AKI, acute kidney injury; AUROC, area under the receiver operating characteristic curve; BMI, body mass index; CI, confidence interval; CIT, cold ischemia time; CKD, chronic kidney disease; DCD, donation after circulatory death; DGF, delayed graft function; eGFR, estimated glomerular filtration rate; mGFR, measured glomerular filtration rate; IGF, immediate graft function; IQR, interquartile range; IRI, ischemia reperfusion injury; KRT, kidney replacement therapy; NGAL, neutrophil gelatinase-associated lipocalin; NPV, negative predictive value; penKid, Proenkephalin A 119-159; PPV, positive predictive value; SCr, Serum Creatinine; SGF, slow graft function.
