# Supplementary material for: Proenkephalin A 119-159 in Kidney Transplantation: A Novel Biomarker for Superior Tracking of Graft Function Trajectories
Source: Transpl Int. 2025 May 22;38:14366. doi: 10.3389/ti.2025.14366 (PMC12138523; doi:10.3389/ti.2025.14366)
Supplement: Supplementary file 1 [file DataSheet1.pdf]

Supplementary material

Table of contents

Table S1: Baseline characteristics Sydney study

Table S2: Diagnostic characteristics to predict outcomes in the Sydney study

Figure S1: Diagnostic performance of changes in proenkephalin A 119-159 levels to predict delayed graft function across subpopulations.

Figure S2: Influence of critical recipient and donor-related factors on proenkephalin A 119-159 levels.

Figure S3: Biomarker trajectories and diagnostic performance of proenkephalin A 119-159 and serum creatinine to predict delayed graft function (Sydney study)

Figure S4: Biomarker trajectory of proenkephalin A 119-159 and serum creatinine to discriminate recovery of graft function (Sydney study).

Figure S5: Biomarker trajectories and diagnostic performance of proenkephalin A 119-159 and serum creatinine to predict 30-day graft function (Sydney study).

Table S1. Baseline characteristics Sydney study

Variable	All<br>N = 60	No DGF<br>N = 34	DGF<br>N = 26	*P*-value
**Recipient**				
Age (years), median (IQR)	58 (45 -65)	50 (41- 60)	64 (56 - 67)	0.01
Sex (female), N (%)	22 (36.7)	13 (38.2)	9 (34.6)	0.77
BMI (kg/m^2), median (IQR)	26.1 (20.9 - 29.3)	26.3 (21.0 - 29.3)	26.2 (20.2 - 29.3)	0.92
Dialysis Vintage (years), median (IQR)	3.0 (1.0 -4.0)	2.0 (0.0 -4.0)	3.5 (1.8 -4.0)	0.04
**Donor**				
Age (years), median [IQR]	54 (44 -61)	54 (41 - 61)	54 (48 - 61)	0.52
Sex (female), N (%)	23 (38.3)	17 (50)	6 (23.1)	0.03
Hypertension, N (%)	24 (40)	10 (29)	14 (53.8)	0.06
Diabetes, N (%)	3 (5)	2 (6)	1 (3.8)	0.72
S-creatinine (mg/dL), median (IQR)	0.83 (0.73 - 1.01)	0.81 (0.72 - 0.93)	0.91 (0.73 - 1.13)	0.15
**Transplant-Related**				
Transplant Modality<br>Living, N (%)<br>Deceased, N (%)	13 (41.7)<br>35 (58.3)	13 (38.2)<br>21 (61.8)	0<br>26 (100)	<0.001
Number of Transplants<br>First, N (%)<br>Retransplants, N (%)	50 (83.3)<br>10 (16.7)	31 (91.2)<br>3 (8.8)	19 (73.1)<br>7 (26.9)	0.06
Cold Ischemia Time (hours), median (IQR)	7.9 (5.0 - 10.4)	6.6 (3.9 - 9.9)	8.9 (7.2 - 10.9)	0.01
Median HLA (A, B, DR) Mismatches (IQR)	4.0 (3.0 - 5.0)	3.5 (3.0 - 5.0)	4.0 (3.0 - 5.3)	0.53
Complement-dependent cytotoxicity (panel reactivity of >30%), N (%)	17 (28.3)	7 (20.6)	10 (38.5)	0.13
Induction Therapy				
Rituximab, N (%)	0	0	0	1
Anti-thymocyte globulin, N (%)	8 (13.3)	2 (5.9)	6 (23.1)	0.05
Interleukin-2 receptor antagonist, N (%)	52 (88.1)	32 (94.1)	20 (76.9)	0.05
Other, N (%)	0	0	0	1
**Short-Term Outcomes**				
Length of Stay (days), median (IQR)	9 (8 - 13)	8.5 (6.0 - 10.3)	12.0 (9.0 - 16.3)	0.001
S-Creatinine at Discharge (mg/dL), median (IQR)	2.93 (1.86 - 5.84)	2.05 (1.35 - 3.01)	6.01 (3.28 - 7.28)	<0.001

BMI, body mass index; DGF, delayed graft function; HLA, human leucocyte antigen; IQR, interquartile range; N, number; penKid, Proenkephalin A 119-159.

Table S2. Diagnostic characteristics to predict outcomes in the Sydney study

Tested outcome	Sensitivity (95%-CI)	Specificity (95%-CI)	PPV	NPV
**DGF**				
penKid > 300 pmol/L	100.0 % (79.6-100.0)	73.9 % (53.5-87.5)	71.4 %	100.0 %
penKid reduction ≤ 50%	100.0 % (75.8-100.0)	65.0 % (43.3-81.9)	63.2 %	100.0 %
**30-d eGFR ≤ 30mL/min**				
penKid > 300 pmol/L	100.0 % (64.5-100.0)	54.8 % (37.8-70.8)	33.3 %	100.0 %
penKid reduction ≤ 50%	100.0 % (56.6-100.0)	48.2 % (30.7-66.1)	26.3 %	100.0 %

CI, confidence interval; DGF, delayed graft function; eGFR, estimated glomerular filtration rate; NPV, negative predictive value; PPV, positive predictive value; penKid, Proenkephalin A 119-159.

Model	N	Events	AUC (95%-CI)
penKid change female	52	24	0.85 [0.75,0.95]
penKid change male	57	13	0.90 [0.82,0.99]
penKid change recipent age ≤ 49 [years]	55	16	0.90 [0.82,0.98]
penKid change recipent age > 49 [years]	54	21	0.84 [0.73,0.95]
penKid change KRT duration pre-Tx ≤ 6 [years]	56	15	0.94 [0.87,1.00]
penKid change KRT duration pre-Tx > 6 [years]	48	22	0.80 [0.68,0.93]
penkid change living donation	33	2	0.97 [0.91,1.03]
penkid change deceased donation	76	35	0.83 [0.74,0.92]
penkid change donor age ≤ 55 [years]	53	17	0.85 [0.74,0.95]
penkid change donor age > 55 [years]	56	20	0.90 [0.82,0.98]
penkid change donor SCr ≤ 0.8 [mg/dL]	62	16	0.89 [0.81,0.97]
penkid change donor SCr > 0.8 [mg/dL]	46	21	0.85 [0.73,0.96]
penkid change CIT ≤ 10.3 [hours]	52	8	0.83 [0.68,0.98]
penkid change CIT > 10.3 [hours]	57	29	0.86 [0.76,0.95]
**penKid change overall**	**109**	**37**	**0.87 [0.81,0.94]**

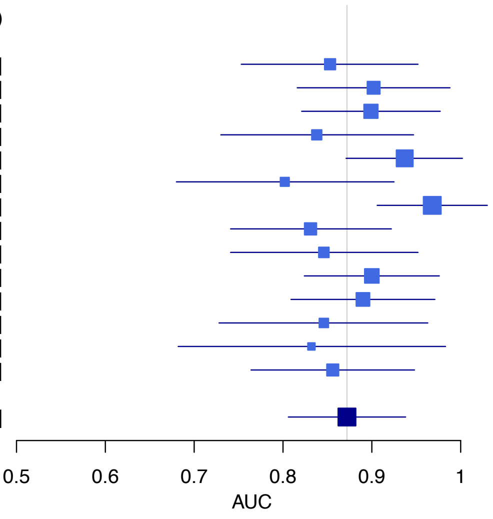

Figure S1. Diagnostic performance of changes in proenkephalin A 119-159 levels to predict delayed graft function across subpopulations. Receiver-operating characteristics analysis for relative penKid change from pre-transplant to first post-transplant day to predict DGF in relevant subgroups based on donor type, critical risk profiles and transplant modalities. For continuous variables the overall mean was used to divide the study cohort in tow group AUC, area under the curve; CIT, cold ischemia time; penKid, Proenkephalin A 119-159; SCr, serum creatinine; Tx, transplant.

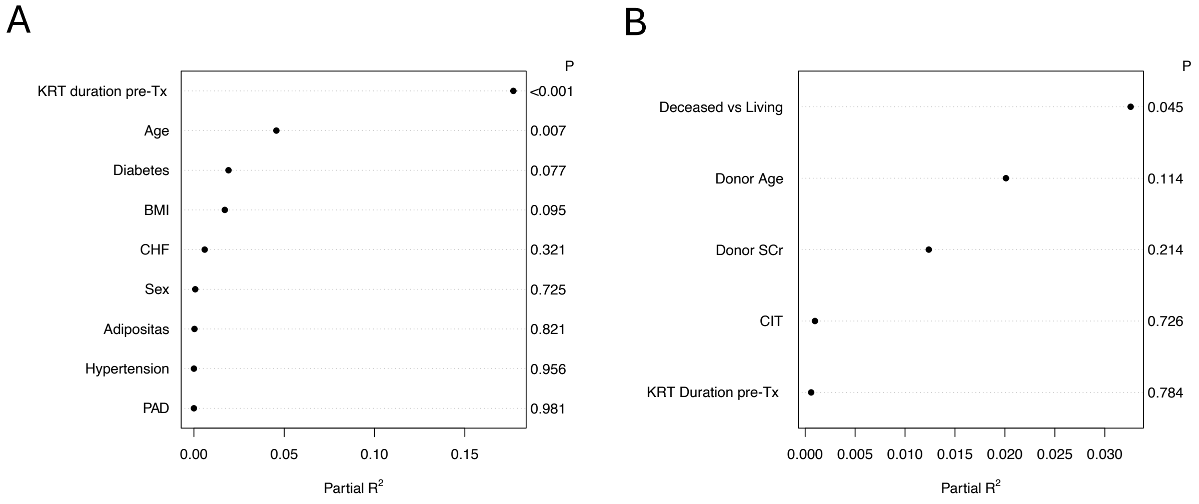

Figure S2. Influence of critical recipient and donor-related factors on proenkephalin A 119-159 levels. Linear regression analyses for absolute penKid levels pre-transplant (A) and penKid changes from pre-transplant to first post-transplant day (B) to assess how critical donor and recipient-related factors known to affect outcomes are associated with penKid blood levels. BMI, body mass index; CHF, congestive heart failure; CIT, cold ischemia time; KRT, kidney replacement therapy; P, p-value; PAD, peripheral artery disease; SCr, serum creatinine; transplant.

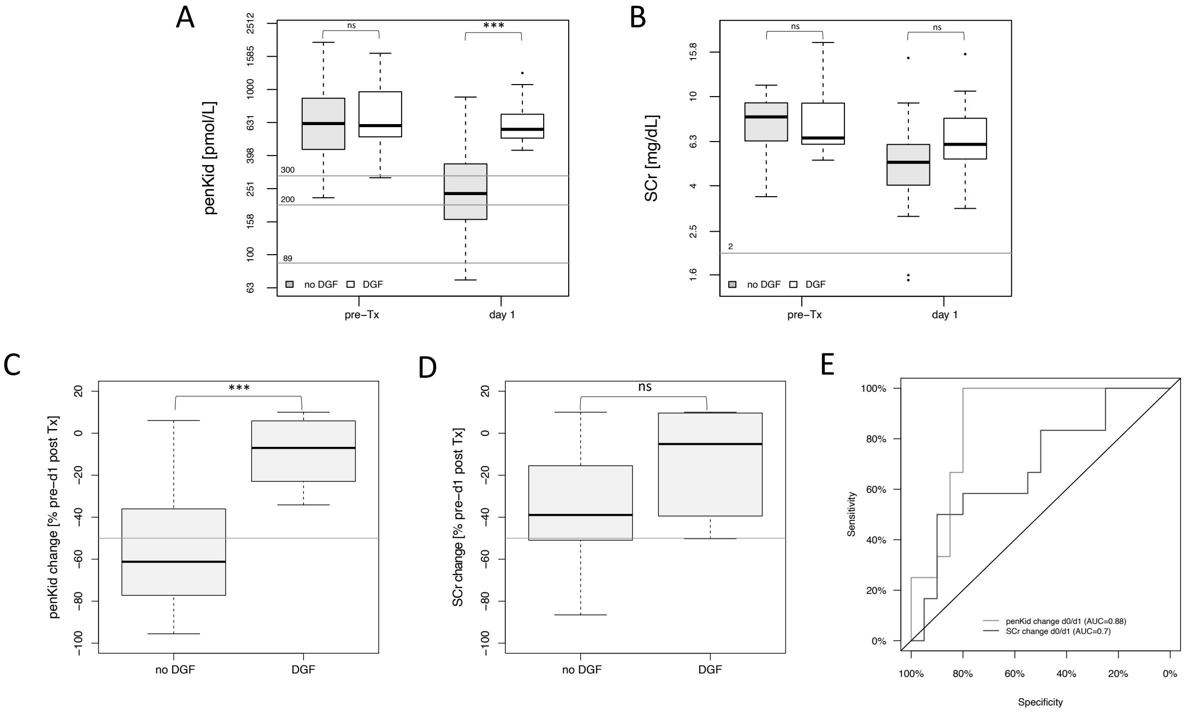

Figure S3. Biomarker trajectories and diagnostic value of proenkephalin A 119-159 and serum creatinine in association with delayed graft function (Sydney study). (A, B) absolute biomarker trajectories prior and first day after kidney transplantation stratified by DGF. (C, D) relative biomarker changes comparing pre-transplant biomarker levels to the respective post-transplant days stratified by DGF. (E) Receiver-operating characteristics analysis for relative biomarker change from pre-transplant to first post-transplant day to predict DGF. DGF: N=26; no DGF: N=34. The grey lines indicate penKid cut-offs at 300 pmol/L,200 pmol/L, and 89 pmol/L (the last being the upper reference limit for healthy individuals) (A, B), or a 50% decrease cut-off compared to pre-transplant biomarker levels (C, D). For SCr, the grey line signifies an SCr of 2mg/dl for orientation. Both y-axes are log-transformed. AUC, area under the curve; d, days; DGF, delayed graft function; penKid, Proenkephalin A 119-159; SCr, serum creatinine; Tx, transplant. *$P \leq 0.05$, **$P \leq 0.01$, ***$P \leq 0.001$. NS, $P > 0.05$.

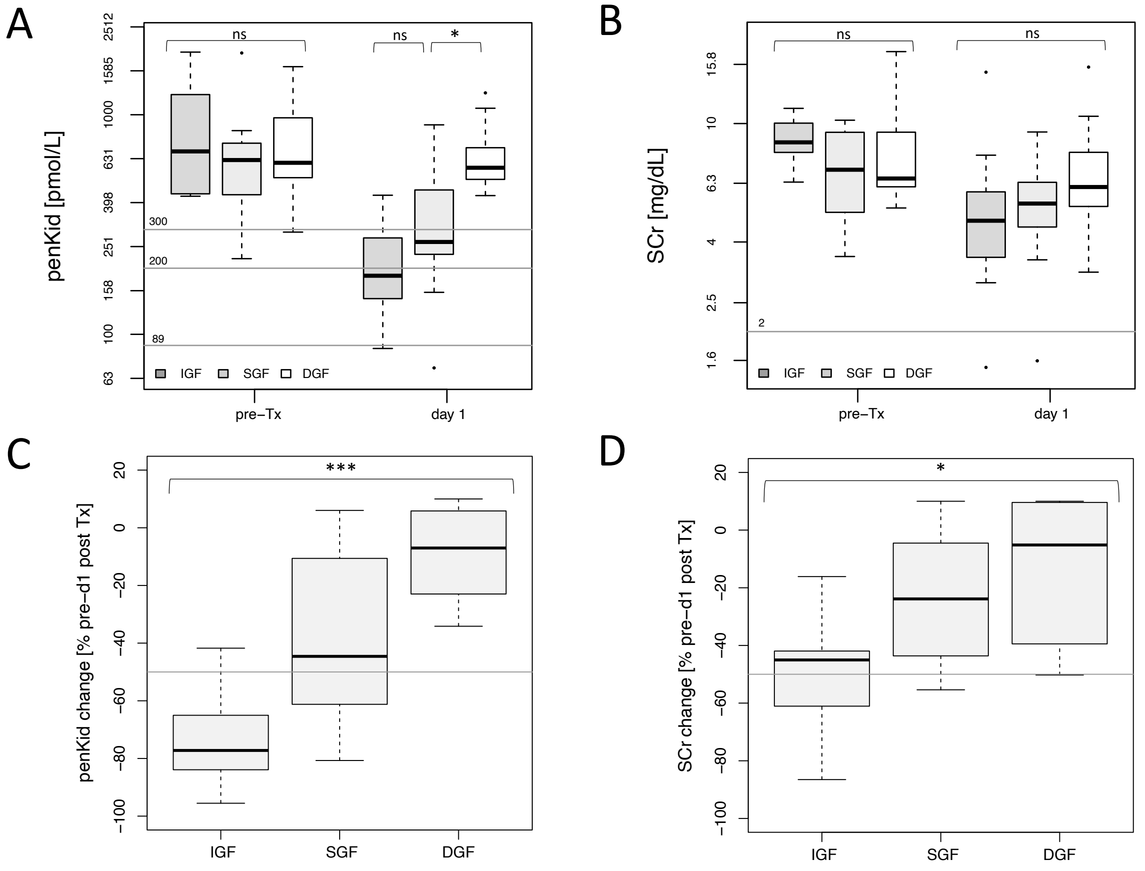

Figure S4. Biomarker trajectory of proenkephalin A 119-159 and serum creatinine to discriminate scenarios of graft function recovery (Sydney study). (A, B) absolute biomarker trajectories prior and first day after kidney transplantation stratified by recovery of graft function. (C, D) relative biomarker changes comparing pre-transplant biomarker levels to the respective post-transplant days stratified by recovery of graft function. IGF: N=12, SGF: N=22, DGF: N=26. The grey lines indicate penKid cut-offs at 300 pmol/L, 200 pmol/L and 89 pmol/L (the last being the upper reference limit for healthy individuals) (A, B), or a 50% decrease cut-off compared to pre-transplant biomarker levels (C, D). For SCr, the grey line signifies an SCr of 2mg/dl for orientation. Both y-axes are log-transformed. d, days; DGF, delayed graft function; IGF, immediate graft function; penKid, Proenkephalin A 119-159; SCr, serum creatinine; SGF, slow graft function; Tx, transplant. *$P \leq 0.05$, **$P \leq 0.01$, ***$P \leq 0.001$. NS, $P > 0.05$.

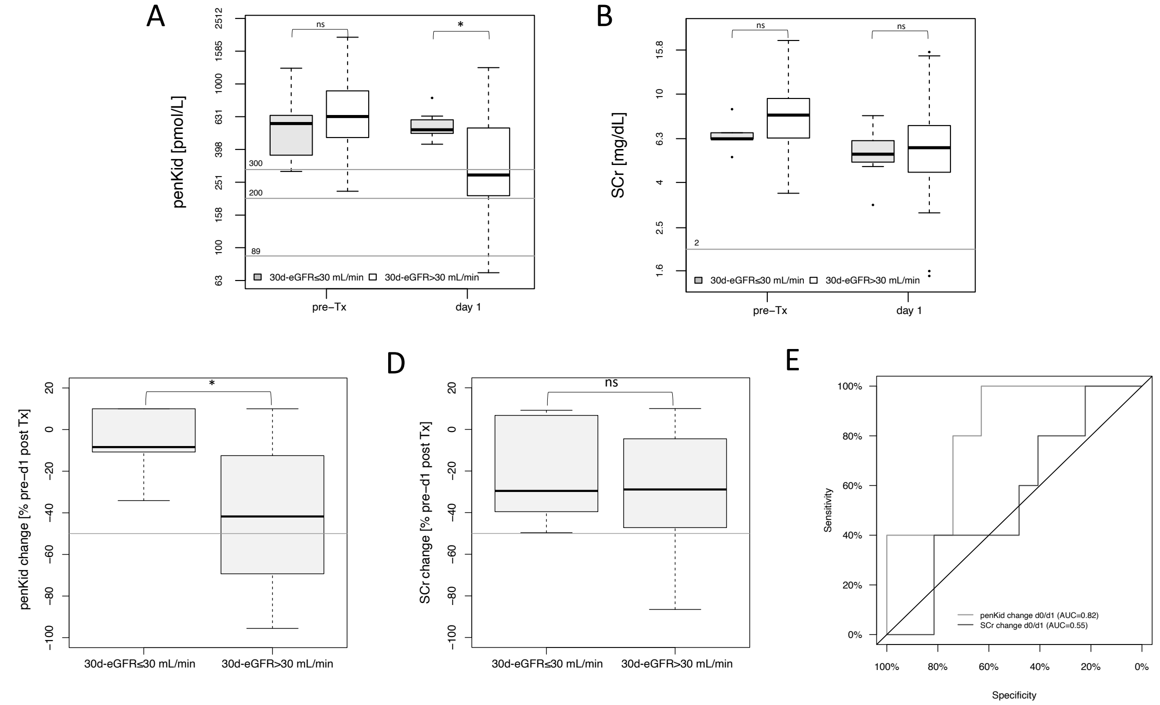

Figure S5. Biomarker trajectories and diagnostic value of proenkephalin A 119-159 and serum creatinine associated with 30-day graft outcome (Sydney study). (A, B) absolute biomarker trajectories prior and first day after kidney transplantation stratified by 30-day graft outcome. (C, D) relative biomarker changes comparing pre-transplant biomarker levels to the respective post-transplant days stratified by 30-day graft outcome. (E) Receiver-operating characteristics analysis for relative biomarker change from pre-transplant to first post-transplant day to predict 30-day graft outcome. 30d-eGFR≤30mL/min: N=14, 30d-eGFR>30mL/min: N=44. The grey lines indicate penKid cut-offs at 300 pmol/L,200 pmol/L, and 89 pmol/L (the last being the upper reference limit for healthy individuals) (A, B), or a 50% decrease cut-off compared to pre-transplant biomarker levels (C, D). For SCr, the grey line signifies an SCr of 2mg/dl for orientation. Both y-axes are log-transformed. AUC, area under the curve; CIT, cold ischemia time; d, days; DGF, delayed graft function; penKid, Proenkephalin A 119-159; SCr, serum creatinine; Tx, transplant. *$P \leq 0.05$, **$P \leq 0.01$, ***$P \leq 0.001$. NS, $P > 0.05$.
